# Altered clot structure in pregnant women who will develop postpartum hemorrhage

**DOI:** 10.1016/j.rpth.2025.102683

**Published:** 2025-01-16

**Authors:** Claire de Moreuil, Brigitte Pan-Petesch, François Anouilh, Dino Mehic, Theresa Schramm, Christoph Friedl, Alisa S. Wolberg, Francis Couturaud, Johanna Gebhart, Cihan Ay, Ingrid Pabinger

**Affiliations:** 1 Univ Brest, INSERM UMR 1304-GETBO, F-CRIN INNOVTE Network, Brest, France; 2 Internal Medicine Department, CHU Brest, Brest, France; 3 Department of Medicine I, Clinical Division of Haematology and Haemostaseology, Medical University of Vienna, Vienna, Austria; 4 Center for Haemophilia Treatment, Haematology, CHU Brest, Brest, France; 5 Univ Brest, University School of Midwifery, Brest, France; 6 Gynecology and Obstetrics Department, CHU Brest, Brest, France; 7 Core Facilities, Medical University of Vienna, Vienna, Austria; 8 Department of Pathology and Laboratory Medicine and UNC Blood Research Center, University of North Carolina at Chapel Hill, North Carolina, USA; 9 Pneumology Department, CHU Brest, Brest, France

Hypercoagulability is a physiological mechanism observed in pregnant women, probably promoted through human evolution to confer a protection against abnormal bleeding at delivery [1]. Indeed, postpartum hemorrhage (PPH) is a main cause of maternal death in women of reproductive age [2]. An inflammatory state is classically seen during pregnancy, along with activation of platelets; increase in circulating coagulation factors, especially in fibrinogen levels; decrease in coagulation inhibitors; and hypofibrinolytic tendency [[3], [4], [5]].

In a nested case-control study, we explored the clot structure of predelivery plasma from pregnant women with and without PPH, in comparison with healthy nonpregnant women. For this purpose, we selected 38 pregnant women included in the “Study of Biological Determinants of Bleeding Postpartum” French cohort study, 19 cases with PPH and a blood loss between 800 and 999 mL at delivery, and 19 pregnant controls without PPH, matched for age, body mass index (BMI), and term and mode of delivery [6,7]. Women with PPH who were included were those for whom 500 μL of frozen plasma was still available after a thorough investigation of their coagulation properties performed in a previous study [8]. No other selection criteria were applied. As controls, we randomly selected 19 healthy nonpregnant women included as healthy controls into the Austrian “Vienna Bleeding Study”, matched for age [9].

For pregnant women, the blood used for the experiment was drawn at entry into the delivery room before delivery. Measurement of blood loss at delivery was performed with a graduated collector bag. PPH etiology was assessed by the midwife or obstetrician in charge of delivery, according to the “4T’s” classification for basic processes leading to PPH (tone, trauma, tissue, and thrombin) [10,11]. All cases were reviewed and adjudicated by 2 independent obstetricians in order to confirm the main etiology of PPH.

Frozen platelet-poor plasma was used for the experiment. Thawed plasma was first spiked with Alexa Fluor 488–conjugated fibrinogen (1.5 mg/mL, diluted in bicarbonate sodium, Thermo Fisher Scientific). A clot was then formed with the spiked plasma from each of the 57 women (150 μL per well), phospholipids (phospholipids 0.5 mM, Rossix; 4 μM final, 6 μL per well), CaCl_2_ (1 M, 10 mM final, 3 μL per well), and tissue factor (Innovin, Siemens; diluted in hepes buffered saline, 0.229 pM final, 3 μL per well) inside an 8-well Lab-Tek II chambered coverglass slide (Thermo Fisher Scientific), in duplicates, as described [12,13]. After incubation, images of the clot were captured with a Zeiss LSM 700 confocal microscope (Core Facilities, MedUni Wien). For each well, a 30-slice Z-stack was captured in 4 different areas, and from this, we determined the median fibrin network density using ImageJ version 1.41o. Fibrin network density was estimated as a percentage by dividing the area covered by fibrin fibers with the area of 1 reading spot. For all steps of the experiment, the researcher was blinded to the plasma attribution group in order to minimize biases. Sample information was revealed only at the end of the study for the statistical analyses. The methods were developed and optimized for reproducibility prior to performing the measurements on the plasma samples studied.

The 38 pregnant women included in the study had a median (IQR) age of 31 (24-35) years and a median (IQR) prepregnancy BMI of 24 (22-29) kg/m^2^. Ten (26.3%) women were smokers, 20 (52.6%) were nulliparous, 2 (5.3%) had gemellar pregnancy, and 2 (5.3%) had abnormal placental insertion. Participants delivered at a median (IQR) term of 40 (38-40) weeks of gestation; 14 (36.8%) delivered vaginally, and 13 (34.2%) had induced delivery.

The 19 healthy nonpregnant women had a median (IQR) age of 31 (25-36) years and a median (IQR) BMI of 22 (20-24) kg/m^2^, and 4 (21.1%) were smokers.

The Table summarizes other coagulation parameters for the 3 groups, including conventional hemostatic tests and thrombin and plasmin generation assay parameters. Median (IQR) predelivery fibrinogen levels were similar between PPH cases and pregnant controls (5.1 [4.6-5.7] g/L vs 5.5 [4.9-5.8] g/L) and, as expected, much higher in pregnant women than in nonpregnant controls (2.9 [2.6-3.3] g/L; *P* <.0001).

**Table:** Coagulation profile of the pregnant women with and without PPH and of the nonpregnant control women.

Hemostatic biomarkers, median (IQR)	PPH cases (*n* = 19)	Pregnant controls (*n* = 19)	*P*^a^	Nonpregnant controls (*n* = 19)	*P*^b^
Blood count parameters					
Hemoglobin, g/dL	11.8 (11.5-13.0)	12.8 (12.2-13.3)	.1	13.3 (12.8-13.5)	.02
Platelets, ×10^9^/L	215 (177-268)	222 (197-291)	.7	268 (239-292)	.09
IPF, ratio	5.1 (3.7-7.0)	4.9 (3.6-7.8)	1.0	NA	
Conventional hemostatic tests					
PT, ratio, %	96 (89-100)	98 (94-100)	.4	97 (90-101)	.7
aPTT, ratio	1.00 (0.95-1.06)	0.99 (0.90-1.04)	.3	0.97 (0.92-1.01)	.4
Fibrinogen, g/L	5.1 (4.6-5.7)	5.5 (4.9-5.8)	.5	2.9 (2.6-3.3)	<.0001
D-dimer, μg/mL	1.6 (1.4-1.9)	1.7 (1.0-2.0)	.7	NA	
Fibrin monomers, μg/mL	6.4 (4.4-8.5)	5.8 (4.1-6.7)	.4	NA	
Thrombin generation assay					
Lag phase, min	13.6 (12.1-15.1)	13.1 (11.6-15.3)	.8	9.1 (7.3-10.8)	<.0001
Thrombin peak, nmol/L	279 (238-420)	350 (301-438)	.3	325 (226-476)	.6
Time to peak, min	23.1 (19.8-26.1)	22.1 (19.6-24.6)	.5	15.1 (12.3-19.3)	.001
Velocity index, nmol/min	29.7 (21.3-56.0)	42.0 (30.8-60.9)	.3	56.8 (26.6-97.3)	0.2
ETP, nM × min	5499 (5228-6041)	5600 (5234-6213)	.5	4087 (3628-4591)	<.0001
Plasmin generation assay					
Lag phase, min	2.7 (2.7-3.0)	2.7 (2.3-3.0)	.2	2.3 (2.0-2.3)	.0001
Plasmin peak, nmol/L	68.1 (55.7-74.5)	61.7 (55.2-71.8)	.7	48.2 (43.2-59.1)	.01
Time to peak, min	7.8 (7.1-8.3)	7.8 (7.3-8.0)	.9	6.3 (6.0-7.1)	<.0001
Velocity index, nmol/min	13.9 (12.0-15.7)	12.8 (10.9-14.2)	.4	12.2 (10.0-14.6)	.5
EPP, nM min	720 (481-1264)	872 (617-1036)	.7	699 (589-904)	.6

aPTT, activated partial thromboplastin time; EPP, endogenous plasmin potential; ETP, endogenous thrombin potential; IPF, immature platelet fraction; NA, not available; PPH, postpartum hemorrhage; PT, prothrombin time.

a Comparison between PPH cases and pregnant controls (Wilcoxon rank sum test).

b Comparison between the 3 groups (Friedman test).

Median (IQR) fibrin network density was reduced in PPH cases compared with matched pregnant controls (30.6% [29.7%-33.6%] vs 34.4% [27.7%-36.0%]; *P* =.04). Healthy nonpregnant controls had a reduced median (IQR) density of fibrin fibers when compared with the 2 groups of pregnant women (28.8% [26.1%-30.1%]; *P* =.003; Figure). The difference in clot density between pregnant and nonpregnant women persisted after adjustment for fibrinogen level (*P* =.02).

**Figure:**
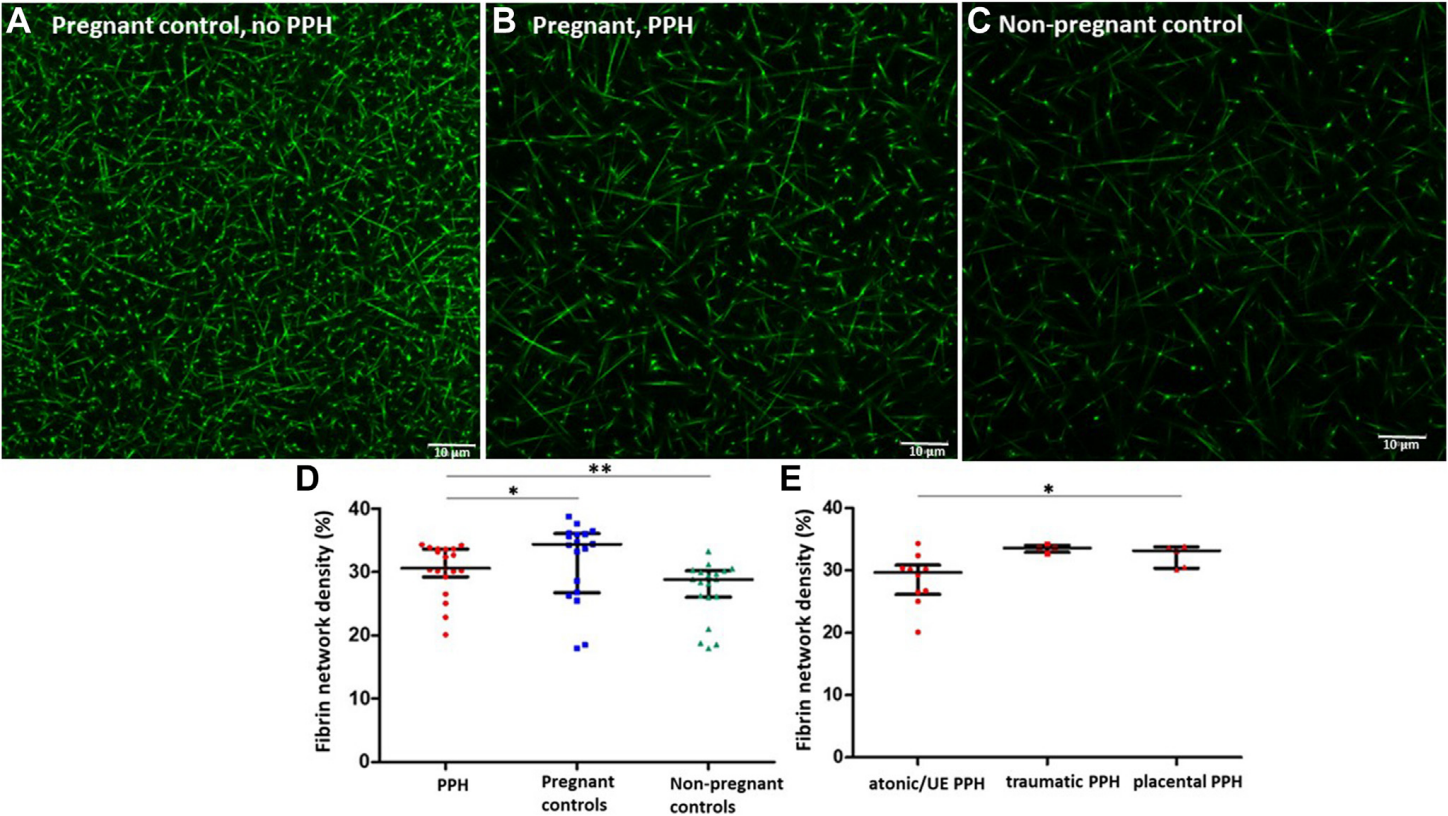
(A–C) Examples of pictures of clots from pregnant women observed with confocal microscopy: a pregnant control (A), a postpartum hemorrhage (PPH) case (B), and a nonpregnant control (C). Fibrin fibers appear in fluorescent green, labeled with Alexa Fluor 488. (D) Comparison of fibrin network density in plasma clots from PPH cases, pregnant controls, and nonpregnant controls observed with confocal microscopy. (E) Comparison of fibrin fiber density in plasma clots from PPH cases according to associated PPH etiology. The scatter dot plots represent median values with IQR. ∗*P* significance ≤ .05; ∗∗*P* significance ≤ .01. UE, undetermined etiology.

Among participants who experienced PPH, 6 (31.6%) were associated with atony, 4 (21.1%) were associated with trauma, 5 (26.3%) were associated with placental defects, and 4 (21.1%) had unknown etiology. The PPH subgroups with uterine atony and unknown etiology had a reduced median (IQR) fibrin network density compared with cases associated with trauma or placental defects (29.7% [25.4%-30.3%] vs 33.6% [33.4%-33.8%] and 32.4% [30.6%-33.2%], respectively; *P* =.04; Figure), despite a higher median predelivery fibrinogen level (*P* =.02).

In conclusion, we showed that clots produced in vitro from the predelivery plasma of pregnant women had a denser fibrin network than clots of nonpregnant healthy women matched for age, even after adjustment for fibrinogen levels. This observation is consistent with an established, pregnancy-associated hypercoagulable state that culminates at the end of third trimester [14]. We also observed reduced fibrin network density in plasma clots of pregnant women who developed later PPH compared with matched pregnant controls, suggesting that PPH is preceded by changes in plasma function that lead to altered clot structure. Notably, reduced fibrin network density is associated with an increased bleeding tendency [15]. Thus, reduced ability to produce an appropriately dense hemostatic fibrin network could contribute to the pathophysiology of PPH. The finding that PPH cases associated with uterine atony or of unknown etiology share a similar fibrin network structure that is less dense than that in cases associated with trauma or placental defects suggests that these groups may benefit the most from prophylactic hemostatic treatments such as tranexamic acid. Obviously, these findings require independent validation on a separate cohort. A recent systematic review and meta-analysis on prophylactic tranexamic acid after cesarean section concluded that this drug may prevent PPH, especially in high-risk pregnant women, which is in line with the positive results of the TRAnexamic Acid for Preventing postpartum hemorrhage after cesarean delivery trial [16,17]. Currently, however, prophylactic use of tranexamic acid at delivery to prevent PPH is not implemented worldwide. By identifying a looser fibrin network in pregnant women who will develop PPH, especially in women diagnosed with uterine atony or with PPH of unknown etiology, our study supports the hypothesis that predelivery hemostatic state is a determinant of PPH risk, and that more tailored prophylactic approaches should be optimized to ensure a delivery without hemorrhagic complications.

The mechanisms leading to a reduced fibrin network density in pregnant individuals who developed PPH are still unknown. Indeed, neither predelivery conventional hemostatic parameters including fibrinogen level nor thrombin or plasmin generation assay parameters differed between PPH cases and matched pregnant controls. Posttranscriptional modifications in fibrinogen may differ between these groups. Alternately, other plasma components are reported to modulate fibrin structure, including albumin and immunoglobulins [15] and may differ between groups. Using an agnostic proteomic analysis, Reitsma et al. [18] identified 77 differentially abundant proteins in plasma from pregnant individuals who did or did not develop PPH. Further studies are needed to characterize the potential contributions of each of these proteins to plasma clot structure and determine if these may yield diagnostic biomarkers or therapeutic targets for preventing PPH.
